# Supplementary material for: Delayed Recovery After Exercise-Induced Pain in People with Chronic Widespread Muscle Pain Related to Cortical Connectivity
Source: Brain Sci. 2024 Oct 30;14(11):1102. doi: 10.3390/brainsci14111102 (PMC11592148; doi:10.3390/brainsci14111102)
Supplement: Supplementary file 1 [file brainsci-14-01102-s001.zip › Supplemental Materials.pdf]

Supplemental Materials

Results

Pain at Rest

Association of SMN Functional Connectivity with Peak Pain at Rest

Greater connectivity of the SMN with bilateral clusters with peak voxels in postcentral gyrus was significantly associated with lower maximum pain at rest after an eccentric exercise. In addition, greater SMN connectivity with a cluster with its peak voxel in left lateral occipital cortex predicted worse pain. Individuals with CPMP had significantly lower resting state connectivity of SMN with the left ($t_{18}=-2.61$, $p=.02$) and right ($t_{18}=-3.59$, $p=.002$) postcentral gyrus clusters than NP, but greater resting state connectivity with the left lateral occipital cortex cluster ($t_{18}=3.15$, $p=.006$; Supplemental Table S1).

Within the SMN, greater FC of left premotor cortex with right premotor cortex ($b=-.005$, $p_{FDR}=.02$) and primary motor cortex ($b=-.006$, $p_{FDR}=.003$), as well as right premotor cortex with primary motor cortex ($b=-.007$, $p_{FDR}=.01$), was associated with lower maximum pain at rest.

Association of Cerebellar Functional Connectivity with Peak Pain at Rest

Greater connectivity of pain-related cerebellar structures with clusters with peak voxels in occipital structures and inferior temporal gyrus was associated with lower maximum pain at rest. However, greater connectivity with a cluster peaking in posterior cingulate cortex

was associated with greater maximum pain. For the clusters peaking in right intracalcarine cortex (t18=-2.85, p=.01), left occipital pole (t18=-2.41, p=.03), right inferior temporal gyrus (t18=-2.12, p=.049), and right lateral occipital cortex (t18=-2.37, p=.03), CPMP individuals had lower resting connectivity than NP. However, they had greater connectivity for the posterior cingulate cluster (t18=2.39, p=.03; Supplemental Table S2).

For associations of intra-cerebellar connectivity with peak pain at rest, we found that greater connectivity between Crus I and Vermis 4 5 was associated with lower pain (b=-.004, t18=-2.72, pFDR=.04).

Association of Frontoparietal Network Connectivity with Peak Pain at Rest

Greater FPN connectivity with clusters centered in right middle temporal gyrus, right angular gyrus, left frontal pole, right frontal pole, left lateral occipital cortex, and right superior frontal gyrus was associated with lower maximum pain at rest. In contrast, greater FPN connectivity with right postcentral gyrus, and left hippocampus was associated with greater maximum pain. CPMP had significantly lower FPN FC than NP for right middle temporal gyrus (t18=-2.73, p=.01), right angular gyrus (t18=-2.79, p=.01), left frontal pole (t18=2.45, p=.02), right frontal pole (t18=-3.72, p=.002), left lateral occipital cortex (t18=-3.23, p=.005), and right superior frontal gyrus (t18=-2.13, p=.047). CPMP had greater FPN FC for right postcentral gyrus (t18=3.36, p=.003) and left hippocampus (t18=2.66, p=.02; Supplemental Table S3).

For associations of intra-FPN connectivity with pain at rest, we found that greater connectivity from left dorsolateral prefrontal cortex to left angular gyrus ($b=-.006$, $t_{18}=-3.11$, $p_{FDR}=.018$), left dorsolateral prefrontal cortex to right angular gyrus ($b=-.004$, $t_{18}=-2.37$, $p_{FDR}=.04$), right dorsolateral prefrontal cortex to right angular gyrus ($b=-.009$, $t_{18}=-4.44$, $p_{FDR}=.0009$), and right dorsolateral prefrontal cortex to left angular gyrus ($b=-.006$, $t_{18}=-3.65$, $p_{FDR}=.003$) were all associated with lower maximum pain. CPMP individuals had lower connectivity between right dorsolateral prefrontal cortex and right angular gyrus than NC ($t_{xx}=$, $p=.04$). No other group differences achieved significance ($p>.07$).

Pain with Movement

Association of SMN Functional Connectivity with Peak Pain with Movement

Greater SMN connectivity with a cluster with its peak voxel in left lateral occipital cortex was associated with greater maximum pain with movement. Mean connectivity with this cluster was significantly higher in the CPMP group than NP ($t_{18}=3.05$, $p=.007$; Supplemental Table S1).

Within the SMN, greater FC of left premotor cortex with right premotor cortex ($b=-.005$, $p_{FDR}=.01$) and primary motor cortex ($b=-.006$, $p_{FDR}=.004$), as well as right premotor cortex with primary motor cortex ($b=-.006$, $p_{FDR}=.01$), was associated with lower maximum pain.

Association of Cerebellar Functional Connectivity with Peak Pain with Movement

Similarly to pain at rest, greater cerebellar connectivity with clusters peaking in left posterior/ventral occipital pole, left lateral occipital pole, and right lingual gyrus was associated with lower maximum pain with movement. CPMP individuals had significantly lower connectivity than NP for each (t18=-2.99, p=.008; t18=-2.43, p=.03; and t18=-3.32, p=.004, respectively; Supplemental Table S2).

No associations of intracerebellar functional connectivity with pain with movement were detected (p > .05).

Association of Frontoparietal Network Connectivity with Peak Pain with Movement

Greater FPN connectivity with clusters centered in right angular gyrus, right middle temporal gyrus, left frontal pole, right frontal pole, left lateral occipital cortex, and right superior frontal gyrus was associated with lower maximum pain with movement. However, the opposite pattern was detected for clusters centered in right postcentral gyrus and left superior temporal gyrus. CPMP had significantly lower FPN connectivity than NP with right angular gyrus (t18=-2.92, p=.009), right middle temporal gyrus (t18=-2.88, p=.01), left frontal pole (t18=-2.96, p=.008), right frontal pole (t18=-3.55, p=.002), left lateral occipital cortex (t18=-3.43, p=.003), and right superior frontal gyrus (t18=-2.37, p=.03). However, they had significantly greater FPN connectivity with right postcentral gyrus (t18=3.56, p=.002) and left superior temporal gyrus (t18=2.48, p=.02; Supplemental Table S3).

For associations of intra-FPN connectivity with pain with movement, greater connectivity between left dorsolateral prefrontal cortex and left (t18=-3.00, p=.01) and right (t18=-3.04,

p=.01) angular gyrus was associated with lower maximum pain. Greater connectivity between right dorsolateral prefrontal cortex and left (t_{18}=-3.24, p=.007) and right (t_{18}=-4.11, p=.002) angular gyrus was also associated with lower maximum pain.

Supplemental Table S1. Clusters where SMN connectivity was associated with maximum pain

Outcome	Cluster Coordinates	Cluster Size	Cluster Regions	Voxels in Region	% Coverage	Cluster p-value (<.05 FDR)	Directionality	NP Connectivity Mean	CPMP Connectivity Mean
Maximum Pain at Rest	-68, -22, 18	317	Left Postcentral Gyrus	108	3	.001	-	.63	.39
			Left Parietal Operculum	56	10				
			Left Supramarginal Gyrus	45	5				
			Left Central Operculum	25	3				
			Left Planum Temporale	17	3				
			Not assigned or less than 1% coverage	66	-				
	-44, -74, 46	203	Left Lateral Occipital Cortex	196	4	.009	+	-.28	-.09
			Not assigned or less than 1% coverage	7	-				
	66, -14, 36	154	Right Postcentral Gyrus	80	2	.02	-	.65	.35
			Right Supramarginal Gyrus	17	2				
			Not assigned or less than 1% coverage	57	-				
Maximum Pain with Movement	-44, -74, 46	233	Left Lateral Occipital Cortex	204	4	.007	+	-.29	-.1
			Left Angular Gyrus	22	2				
			Not assigned or less than 1% coverage	7	-				

CWMP – chronic widespread muscle pain; NP – no pain/painfree

Supplemental Table S2. Clusters where cerebellar connectivity was associated with maximum pain

Outcome	Cluster Coordinates	Cluster Size	Cluster Regions	Voxels in Region	% Coverage	Cluster p-value (<.05 FDR)	Directionality	NP Connectivity Mean	CPMP Connectivity Mean
Maximum Pain at Rest	24, -60, 0	2097	Right Intracalcarine Cortex	373	50	<.0001	-	.17	.037
			Left Occipital Pole	235	9				
			Right Lingual Gyrus	217	13				
			Right Occipital Pole	189	8				
			Left Intracalcarine Cortex	146	23				
			Left Lateral Occipital Cortex	112	2				
			Left Cuneus	76	15				
			Right Supracalcarine Cortex	73	51				
			Right cuneus	71	11				
			Left Lingual Gyrus	62	4				
			Left Supracalcarine Cortex	18	25				
			Not assigned or less than 1% coverage	525	-				
	-8, -18, 30	160	Posterior Cingulate Gyrus	67	3	.02	+	-.097	.023
			Anterior Cingulate Gyrus	15	1				
			Not assigned or less than 1% coverage	78	-				
	-12, -90, -16	157	Left Occipital Pole	88	3	.02	-	.17	.05
			Left Lingual Gyrus	23	2				
			Left Occipital Fusiform Gyrus	16	2				
			Not assigned or less than 1% coverage	30	-				
	54, -40, -26	151	Right Inferior Temporal Gyrus (posterior)	68	7	.02	-	.099	.009

			Right Inferior Temporal Gyrus (temporooccipital)	31	4				
			Not assigned or less than 1% coverage	52	-				
	40, -80, 8	121	Right Lateral Occipital Cortex (inferior)	87	.4	.03	-	.13	.006
			Right Lateral Occipital Cortex (superior)	34	1				
Maximum Pain with Movement	-18, -82, 20	961	Left Occipital Pole	125	5	<.0001	-	.18	.03
			Left Intracalcarine Cortex	97	15				
			Left Lateral Occipital Cortex	88	2				
			Right Occipital Pole	85	3				
			Right Intracalcarine Cortex	83	11				
			Left Cuneus	62	12				
			Right Supracalcarine Cortex	56	39				
			Right Cuneus	42	7				
			Left Lingual Gyrus	20	1				
			Left Supracalcarine Cortex	12	16				
			Not assigned or less than 1% coverage	291	-				
	24, -60, 0	166	Right Lingual Gyrus	106	6	.002	-	.16	.028
			Right Intracalcarine Cortex	27	4				
			Not assigned or less than 1% coverage	33	-				
	-16, -96, -16	161	Left Occipital Pole	118	4	.02	-	.14	.025
			Left Occipital Fusiform Gyrus	19	2				
			Not assigned or less than 1% coverage	24	-				

CWMP – chronic widespread muscle pain; NP – no pain/painfree

Supplemental Table S3. Clusters where FPN connectivity was associated with maximum pain

Outcome	Cluster Coordinates	Cluster Size	Cluster Regions	Voxels in Region	% Coverage	Cluster p-value (<.05 FDR)	Directionality	NP Connectivity Mean	CPMP Connectivity Mean
Maximum Pain at Rest	66, -24, -28	344	Right Middle Temporal Gyrus	180	13	.0003	-	.53	.27
			Right Inferior Temporal Gyrus	118	12				
			Not assigned or less than 1% coverage	46	-				
	46, -56, 38	328	Right Angular Gyrus	259	18	.0003	-	.76	.53
			Right Lateral Occipital Cortex	57	1				
			Not assigned or less than 1% coverage	12	-				
	54, -22, 26	308	Right Postcentral Gyrus	152	5	.0003	+	-.28	-.1
			Right Supramarginal Gyrus	53	7				
			Not assigned or less than 1% coverage	103	-				
	-30, 58, -12	222	Left Frontal Pole	221	3	.002	-	.51	.32
			Not assigned or less than 1% coverage						
	-54, -62, 44	167	Left Lateral Occipital Cortex	111	2	.009	-	.7	.49
			Left Angular Gyrus	29	3				
			Left Supramarginal Gyrus	14	1				

			Not assigned or less than 1% coverage	13	-				
	30, 16, 62	154	Right Superior Frontal Gyrus	98	4	.01	-	.51	.28
			Right Middle Frontal Gyrus	24	1				
			Not assigned or less than 1% coverage	32	-				
	-32, -18, -16	130	Left Hippocampus	95	12	.02	+	-.12	.019
			Left Amygdala	10	3				
			Not assigned or less than 1% coverage	25	-				
Maximum Pain with Movement	54, -56, 38	556	Right Angular Gyrus	391	27	<.0001	-	.7	.47
			Right Lateral Occipital Cortex	139	3				
			Not assigned or less than 1% coverage	26	-				
	68, -30, -18	409	Right Middle Temporal Gyrus	234	17	<.0001	-	.52	.25
			Right Inferior Temporal Gyrus	119	13				
			Not assigned or less than 1% coverage	56	-				
	-30, 60, -12	256	Left Frontal Pole	256	4	.001	-	.53	.32
	-54, -62, 44	179	Left Lateral Occipital Cortex	127	3	.006	-	.74	.52
			Left Angular Gyrus	37	4				
			Not assigned or less than 1% coverage	15	-				
	30, 62, -12	125	Right Frontal Pole	125	2	.03	-	.48	.23
	64, -12, 42	110	Right Postcentral Gyrus	58		.04	+	-.25	-.025
			Right Supramarginal Gyrus	8	1				
			Not assigned or less than 1% coverage	44	-				

26, 18, 64	105	Right Superior Frontal Gyrus	58	2	.04	-	.52	.27
		Right Middle Frontal Gyrus	26	1				
		Not assigned or less than 1% coverage	21	-				
24, 60, 2	99	Right Frontal Pole	99	1	.04	-	.48	.36
-48, -38, 6	99	Left Superior Temporal Gyrus	5	1	.04	+	-.045	.097
		Not assigned or less than 1% coverage	94	-				

CWMP – chronic widespread muscle pain; NP – no pain/painfree

Supplemental Table S4. Clusters where SMN connectivity was associated with <u>time</u> to maximum pain and recovery time

Outcome	Cluster Coordinates	Cluster Size	Cluster Regions	Voxels in Region	% Coverage	Cluster p-value (<.05 FDR)	Directionality	NP Connectivity Mean	CWMP Connectivity Mean
Time to Maximum Pain at Rest	16, 64, -8	172	Right Frontal Pole	148	2	.03	+	-.26	-.07
		24	Not assigned or less than 1% coverage	24	-				
Recovery Time for Pain at Rest	-24, -28, 72	298	Left Postcentral Gyrus	144	4	.0008	-	.62	.36
			Left Superior Parietal Lobule	71	5				
			Left Precentral Gyrus	58	1				
			Not assigned or less than 1% coverage	25	-				
	-48, -68, 44	243	Left Lateral Occipital Cortex	237	1	.0025	+	-.28	-.09
			Not assigned or less than 1% coverage	6	-				
	-68, -22, 18	239	Left Postcentral Gyrus	125	3	.0025	-	.63	.37
			Left Supramarginal Gyrus	35	4				
			Left Parietal Operculum	11	2				
			Left Central Operculum	6	1				
			Not assigned or less than 1% coverage	62	-				
	-14, 68, -2	183	Left Frontal Pole	183	3	.008	+	-.28	-.04
	16, 66, -10	164	Right Frontal Pole	164	2	.01	+	-.26	-.05
	16, 30, 58	148	Right Superior Frontal Gyrus	140	5	.01	+	-.23	-.04
			Not assigned or less than 1% coverage	8	-				
	66, -12, 36	140	Right Postcentral Gyrus	89	3	.01	-	.68	.36

			Not assigned or less than 1% coverage	51	-				
	16, -34, 78	116	Right Postcentral Gyrus	83	3	.03	-	.58	.34
			Not assigned or less than 1% coverage	33	-				
Time to Maximum Pain with Movement	8, -30, 52	270	Right Precentral Gyrus	114	3	.002	-	.52	.34
			Right Supplemental Motor Cortex	47	7				
			Anterior Cingulate Cortex	30	1				
			Right Postcentral Gyrus	22	1				
			Left Supplemental Motor Area	7	1				
			Not assigned or less than 1% coverage	50	-				
	-14, -40, 50	201	Left Postcentral Gyrus	85	2	.006	-	.37	.20
			Precuneous	49	1				
			Not assigned or less than 1% coverage	67	-				
	18, -46, 54	134	Precuneous	33	1	.025	-	.38	.23
			Right Postcentral Gyrus	21	1				
			Not assigned or less than 1% coverage	80	-				
Recovery Time for Pain with Movement	-42, -72, 50	372	Left Lateral Occipital Cortex	342	7	.0004	+	-.28	-.095
			Left Angular Gyrus	17	2				
			Not assigned or less than 1% coverage	13	-				
	-68, -22, 16	317	Left Postcentral Gyrus	117	3	.0006	-	.65	.42
			Left Parietal Operculum	45	8				
			Left Supramarginal Gyrus	42	4				

		Left Planum Temporale	37	7				
		Left Central Operculum	28	3				
		Not assigned or less than 1% coverage	48	-				
66, -12, 36	298	Right Postcentral Gyrus	156	5	.0006	-	.63	.36
		Right Supramarginal Gyrus	68	8				
		Not assigned or less than 1% coverage	74	-				
-30, -28, 74	133	Left Postcentral Gyrus	68	2	.03	-	.63	.38
		Left Precentral Gyrus	50	1				
		Not assigned or less than 1% coverage	15	-				
16, 30, 58	114	Right Superior Frontal Gyrus	111	4	.049	+	-.26	-.086
		Not assigned or less than 1% coverage	3	-				

CWMP – chronic widespread muscle pain; NP – no pain/painfree

Supplemental Table S5. Clusters where cerebellar connectivity was associated with time to maximum pain and recovery time

Outcome	Cluster Coordinates	Cluster Size	Cluster Regions	Voxels in Region	% Coverage	Cluster p-value (<.05 FDR)	Directionality	NP Connectivity Mean	CWMP Connectivity Mean
Recovery Time for Pain at Rest	-20, -82, 18	1489	Right Occipital Pole	192	8	<.0001	-	.15	.012
			Right Lateral Occipital Cortex	176	9				
			Right Lingual Gyrus	137	8				
			Left Occipital Pole	127	5				

			Left Lateral Occipital Cortex	107	2				
			Right Lateral Occipital Cortex	56	1				
			Right Intracalcarine Cortex	48	6				
			Right Cuneus	36	6				
			Right Supracalcarine Cortex	25	17				
			Left Cuneus	16	3				
			Left Intracalcarine Cortex	8	1				
			Right Temporal Occipital Fusiform Cortex	6	1				
			Left Supracalcarine Cortex	2	3				
			Not assigned or less than 1% coverage	553	-				
Recovery Time for Pain with Movement	-20, -82, 20	1812	Right Lingual Gyrus	248	14	<.0001	-	.17	.028
			Right Intracalcarine Cortex	213	12				
			Right Occipital Pole	183	10				
			Left Occipital Pole	140	8				
			Left Lateral Occipital Cortex	124	7				
			Right Cuneus	73	4				
			Left Cuneus	55	3				
			Right Lateral Occipital Cortex	47	3				
			Right Supracalcarine Cortex	44	2				
			Left Intracalcarine Cortex	27	1				
			Right Lateral Occipital Cortex	20	1				
			Not assigned or less than 1% coverage	638	-				

CWMP – chronic widespread muscle pain; NP – no pain/painfree

Supplemental Table S6. Clusters where frontoparietal network connectivity was associated with time to recovery

Outcome	Cluster Coordinates	Cluster Size	Cluster Regions	Voxels in Region	% Coverage	Cluster p-value (<.05 FDR)	Directionality	NP Connectivity Mean	CWMP Connectivity Mean
Pain at Rest	66, -12, 38	557	Right Postcentral Gyrus	235	7	<.0001	+	-.26	-.08
			Right Precentral Gyrus	130	3				
			Right Supramarginal Gyrus	25	3				
			Not labeled or less than 1% coverage	167	-				
	-48, -74, 36	205	Left Lateral Occipital Cortex	205	4	.006	-	.57	.35
Pain with Movement	68, -14, 28	424	Right Postcentral Gyrus	182	6	<.0001	+		
			Right Precentral Gyrus	82	2				
			Right Supramarginal Gyrus	15	2				
	-46, -76, 38	222	Left Lateral Occipital Cortex	214	4	.004	-		
			Left Angular Gyrus	5	1				
			Not labeled or less than 1% coverage	3	-				
	58, -58, 40	137	Right Angular Gyrus	101	7	.03	-		
			Left Lateral Occipital Cortex	27	1				
			Not labeled or less than 1% coverage	9	-				

CWMP – chronic widespread muscle pain; NP – no pain/painfree
