# Supplementary material for: Delayed Recovery After Exercise-Induced Pain in People with Chronic Widespread Muscle Pain Related to Cortical Connectivity
Source: Brain Sci. 2024 Oct 30;14(11):1102. doi: 10.3390/brainsci14111102 (PMC11592148; doi:10.3390/brainsci14111102)
Supplement: Supplementary file 1 [file brainsci-14-01102-s001.zip › Supplemental-material-measures.pdf]

Supplemental material – Measures

<u>Pain-related psychological questionnaires</u>:

The Pain Catastrophizing Scale (PCS) uses a 13-item, 5-point Likert scale with higher scores indicating elevated levels of catastrophizing [10]. Items relate to rumination (eg, “I keep thinking about how much it hurts”), magnification (eg, “I wonder whether something serious may happen”), and helplessness (eg, “It's awful and I feel that it overwhelms me”). These are scored from 0 (“not at all”) to 4 (“all the time”). Total scores range from 0 to 52 points.[10]

The Brief Resilience Scale (BRS) is a 6-item self-report measure that assesses the ability to recover from stressful or difficult life events.[11] Items on the BRS are scored on a 5-point Likert Scale. The total BRS score (range 1–5) is an average of all items (after reverse coding 3 items) with higher scores indicating more resilient individuals.[11]

The Pain Resilience Scale (PRS) is a pain specific measure of resilience with cognitive, affective and perseverance components. Validity is supported by its relationship to pain coping measures, and prediction of individual differences in response to painful stimulation. The measure has 14 items over three scales: behavioral perseverance, cognitive, and affective positivity. This instrument is scored on a 5-point Likert scale; the total score for PRS ranges from 0 to 56. Higher scores indicate higher levels of pain resilience.[12]

<u>Daily Pain Intensity Measures</u>

Participants completed daily pain diaries using the Research Electronic Data Capture (REDCap) system beginning the day after Session 1 and continued for fourteen days. These daily pain measures were used to: 1) report the pain associated with DOMS after induction and 2) evaluate the rate of recovery. The survey asked participants to rate the pain intensity in the arm at rest ("when your arm is by your side" and with movement ("when you bend and straighten your elbow") using a 100mm visual analogue scale (VAS) anchored at one end with "no pain" and at the other with "worst pain imaginable". Diaries were delivered to participants at the same time each day with a reminder notification to complete the diary.

Structural and functional MRI acquisition

Anatomical and functional (fMRI) data were collected from each participant prior to DOMS induction using a research-dedication Philips Elition whole-body 3T scanner. Sequentially, participants had a survey/reference and high-resolution 3D anatomical MRI scan performed first, followed by a resting-state fMRI scan was then completed. High-resolution structural brain images were collected using a 3-dimensional (3D) T1-weighted magnetization-prepared rapid gradient-echo (MP-RAGE) sequence with a field-of-view (FOV)=257 mm (FH) x 257 mm (AP) x 180 mm (RL), voxel wise resolution= .67 x .67 x .70 mm, TR=1.1 ms, TE=5.1 ms, FA=8°. Acquisition time was 5.63 minutes. Resting state functional data were collected in the transaxial orientation using an EPI sequence with FOV=225 mm (FH) x 225 mm (AP) x 144 mm (RL), voxel-wise resolution = 1.17 mm X 1.17 mm X 2 mm; gap thickness = 0 mm; multiband factor = 3; TR/TE/FA = 3s/30ms/75º. Acquisition time was 11.8 minutes

Experimental-induced pain protocol

First, maximum voluntary contraction (MVC) of elbow flexion strength was tested using a Biodex System Isokinetic Dynamometer (Biodex Medical Systems, Shirley, NY). Participants were seated and secured within the testing apparatus with a stabilizing strap attached proximal to the elbow joint. The device was locked into place (at 90^0 elbow flexion) and the subject was instructed to build up force against the grip handle of the machine to bend the elbow. Participants performed three repetitions of the strength test.

To perform the dynamic fatiguing exercise bout, the participant was seated in the Biodex dynamometer and secured with belts to prevent trunk movement. Participants performed 3 sets of 15 repetitions at a speed of $60°.s^{-1}$ of eccentric (elbow straightening/biceps lengthening) exercises intended to induce DOMS at the biceps. They then had isometric strength reassessed. A post-set isometric MVIC of 50% or less was required to consider the exercise finished. If the torque had not decreased by 50% or greater, participants performed a maximum of three additional sets of 15 repetitions.
